# Trichostatin A Targets the Mitochondrial Respiratory Chain, Increasing Mitochondrial Reactive Oxygen Species Production to Trigger Apoptosis in Human Breast Cancer Cells

**DOI:** 10.1371/journal.pone.0091610

**Published:** 2014-03-13

**Authors:** Shujuan Sun, Yingyan Han, Jia Liu, Yong Fang, Yuan Tian, Jianfeng Zhou, Ding Ma, Peng Wu

**Affiliations:** Cancer Biology Research Center, Tongji Hospital, Tongji Medical College, Huazhong University of Science and Technology, Wuhan, China; Roswell Park Cancer Institute, United States of America

## Abstract

**Aim:**

Histone deacetylase inhibitors (HDACIs)-based therapies have stimulated interest via their anti-tumor activities, including apoptosis induction, cell cycle arrest, cell differentiation, and autophagy. However, the mechanisms of HDACI-associated anti-tumor activity are not yet clearly defined. The aim of this study was to explore the key events of Trichostatin A (TSA), a classic HDACI agent, against breast cancer cells.

**Methods:**

The MCF-7, MDA-MB-231 and MCF-10A cell lines were evaluated with colony-forming and cell viability assays. Apoptosis and cell cycle distribution were detected by flow cytometry. Mitochondrial function was measured with biochemical assays, flow cytometry and transmission electron microscopy.

**Results:**

TSA inhibited breast cancer cell viability and proliferation, without affecting MCF-10A cell. TSA-induced breast cancer cell apoptosis was initiated by G2-M arrest and depended on mitochondrial reactive oxygen species (ROS) produced subsequent to reduced mitochondrial respiratory chain activity. The enhanced mitochondrial ROS production and apoptosis in cancer cells were markedly attenuated by antioxidants, such as N-acetyl cysteine (NAC), reduced glutathione (GSH) and Vitamin C.

**Conclusion:**

The present study demonstrated that TSA-induced cell death by arresting cell cycle in G2-M phase and was dependent on production of mitochondria-derived ROS, which was derived from impaired mitochondrial respiratory chain.

## Introduction

Despite growing advances in targeted therapies and screening techniques, breast cancer remains a leading cause of malignant mortality in women[1]. The onset and progression of breast cancer involve both genetic and epigenetic changes, and the latter are potentially reversible processes[2]. Histone deacetylase inhibitors (HDACIs), either alone or in combination with other agents, have consistently shown promise in clinical trials in the treatment of breast cancer[3].

Several investigations have revealed that Trichostatin A (TSA), which was originally identified as a fungicidal antibiotic and was a classic HDACI agent[4], exhibited breast cancer cell toxicity in a dose-dependent manner both in vitro and in vivo[5]–[7]. HDACIs majorly altered the expression of specific genes that were important for cellular biological outcomes and mitosis in order to promote cancer cell death, but HDACIs have not been rigorously proven to primarily target transcription to mediate their biological effects[8]. The intrinsic apoptosis pathway is an essential event involved in the anti-tumor activity of TSA. Vorinostat and TSA have been shown to increase the transcription of BH3-only Bcl-2 family genes[9] and, as demonstrated, to disrupt mitochondrial membrane potential, induce the release of cytochrome c from the mitochondrial inter-membrane space to the cytoplasm and activate caspase-9 [10], [11]. Reactive oxygen species (ROS) play an important role in apoptosis, and HDACIs promote ROS production, thus promoting apoptosis[12]–[15]. ROS is a collective phrase that describes a variety of molecules and free radicals, including hydrogen peroxide (H_2_O_2_) and hydroxyl radicals (HO⋅)[16]. The mitochondrial respiratory chain is the vital site of ROS production, and complexes I and III have been suggested to be the major sources of ROS[17], [18].

However, previous studies have not adequately elucidated the mechanisms that underlie TSA activity and selectivity. Thus, the goal of our study was to explore how TSA inhibited breast cancer cell viability and to provide supporting evidence for clinical combination therapies. To this end, we used two breast cancer cell lines, MCF-7 and MDA-MB-231, and the MCF-10A normal mammary epithelial cell line. With this study, we are the first to determine that TSA-mediated apoptosis in breast cancer cells was initiated by G2/M arrest and dependent on elevated mitochondrial-derived ROS derived from the impaired mitochondrial respiratory chain.

## Materials and Methods

### Cell lines and culture

MCF-10A, MCF-7 and MDA-MB-231 cells were purchased from the American Type Culture Collection (ATCC, Manassas, VA, USA). MCF-10A cells were cultured in DMEM/F12 supplemented with 20 ng/ml of epidermal growth factor, 100 ng/ml of cholera toxin, 0.01 mg/ml of insulin, 500 ng/ml of hydrocortisone, and 5% horse serum. MDA-MB-231 cells were cultured in Leibovitz's L-15 Medium. MCF-7 cells were cultured in DMEM supplemented with 0.01 mg/ml of insulin. Each cell line was maintained at 37 °C in a humidified atmosphere with 5% CO_2_.

### MTT assay

A total of 5,000 cells in the log phase of growth were plated into 96-well Costar plates. After 24 h incubation, the cells were exposed to different doses of TSA (T8552, Sigma) and incubated for the indicated time. The cells were assayed by MTT as described previously[19].

### Colony-forming assay

Cells were seeded into 6-cm dishes in triplicate at a density of 1000 cells per dish. The cells were exposed to TSA for 14 d in a humidified incubator at 37°C. Colonies were fixed with 4% paraformaldehyde, stained with 0.5% crystal violet and counted.

### Western blot analysis

Protein levels were analyzed in whole cell lysates that were obtained with RIPA lysis buffer (Beyotime, China), and 50 μg of each sample were resolved on a SDS polyacrylamide gel. Gels were analyzed by immunoblotting with antibodies against Cytochrome C, COX IV, Bcl-xL, Bcl-2, NDUFS1, NDUFS6, UQCRFS1, CYC1 (10993-1-AP, 11242-1-AP, 10783-1-AP, 12789-1-AP, 19532-1-AP, 12444-1-AP, 14417-1-AP, 18443-1-AP, 10242-1-AP, respectively; Proteintech; 1∶1000 dilutions), GAPDH, Bax, cleaved PARP, CyclinB1, Phospho-cdc2(Thr161) (2118S, 5023, 9541, 4138, 9114 respectively; Cell Signaling; 1∶1000 dilutions), H3P (ab5176, 1∶2000 dilutions) and acetylated histone H3 (06-599; Millipore; 1∶1000 dilutions). Immunoblotting analysis was performed with Enhanced Chemiluminescence (ECL) Western Blotting Detection reagents (Pierce).

### Apoptosis assay

The cells were prepared as indicated. After harvesting and washing once in PBS, the cells were stained with the Annexin V-FITC/PI Apoptosis Detection Kit (KeyGen Bio-tech) according to the manufacturer's instructions, and the analysis was performed on a flow cytometer (BD).

### Cell cycle distribution analysis

The cells were treated as indicated. The cells were collected, fixed in 70% ethanol and stored at −20°C overnight. For analysis, the samples were washed once in PBS and stained with propidium iodide (PI; 50 μg/mL) and RNaseA (2 μg/mL) for 15 minutes at room temperature. The stained cells were analyzed on a flow cytometer (BD).

### Cytochrome C release assay

Cellular fractionation into cytosolic and mitochondria fractions was performed with the Mitochondria Isolation Kit for Cultured Cells (89874; Thermo Scientific). Cytochrome c release was monitored by immunoblotting of both the supernatant and mitochondrial fractions.

### Measurement of mitochondrial superoxide

Mitochondria-mediated ROS generation was detected with the mitochondrial superoxide indicator MitoSOX-Red (Invitrogen). The cells were harvested, washed twice in PBS and incubated with 5 μmol/L of MitoSOX-Red for 15 min at 37°C, followed by analysis on a flow cytometer (BD; 10,000 cells/sample).

### Oxygen consumption

Oxygen consumption rate (OCR) was measured at 37°C using XF Cell Mito Stress Test Kit (Seahorse Bioscience, Part # 101706-100) and XF96 extracellular analyzer (Seahorse Bioscience). 6000 Cells were seeded in 96-well plates. After 6 hr, cells were treated with TSA for 18 hr and loaded into the machine for O_2_ concentration determinations. Cells were sequentially exposed to oligomycin (1 μM), carbonylcyanide p-trifluoromethoxyphenylhydrazone (FCCP; 300 nM) and rotenone (100 nM) and antimycin (100 nM). After each injection, OCR was measured for 3 min, the medium was mixed and again measured for 3 min. Every point represents average of 5 different wells. OCR was calculated normalized to the cell number.

### Mitochondrial membrane potential assay and cellular ATP measurement

Cells were treated, collected and then loaded with 50 nmol/L of the fluorescent potential-dependent indicator tetramethylrhodamine ethyl ester (TMRE; Molecular Probes) for 20 min at 37°C. The fluorescence intensity was detected on a flow cytometer (BD; 10,000 cells/sample). Intracellular ATP levels were determined with a commercially available Colorimetric ATP Assay Kit (Beyotime, Shanghai, China) according to the manufacturer's instructions. ATP levels were normalized to the protein levels.

### Enzymatic activity of the electron-transport-chain components

Complex I, complex II, complex IV and complex V activity were assayed with the Complex Human Enzyme Activity Microplate Assay Kit (Abcam) according to the manufacturer's instructions. Complex III activity was assayed with the Mitochondrial Complex III Activity Detection kit (GENMED, China).

### Transmission electron microscopy

Cells were collected and fixed with 2.5% glutaraldehyde in 0.1 mol/L sodium cacodylate buffer and then post-fixed with 2% osmium tetroxide in 0.1 mol/L sodium cacodylate buffer plus 0.3% potassium ferrocyanide. The cells were stained with 4% aqueous uranyl acetate, dehydrated, infiltrated and embedded in epoxyresin. Ultrathin sections (80 nm) were cut and imaged with an aFEI Tecnai G^2^ electron microscope.

### Statistical analysis

The statistical analysis was performed with the SPSS 16.0 software package. Statistical comparisons between two groups were conducted with the two-tailed Student's t-test or, when not normally distributed, the Mann-Whitney U-test. A one-way ANOVA was used for multiple-group comparisons. A *P*-value<0.05 was defined as statistically significant.

## Results

### Effects of TSA on breast cancer cell viability and proliferation

**Figure 1:**
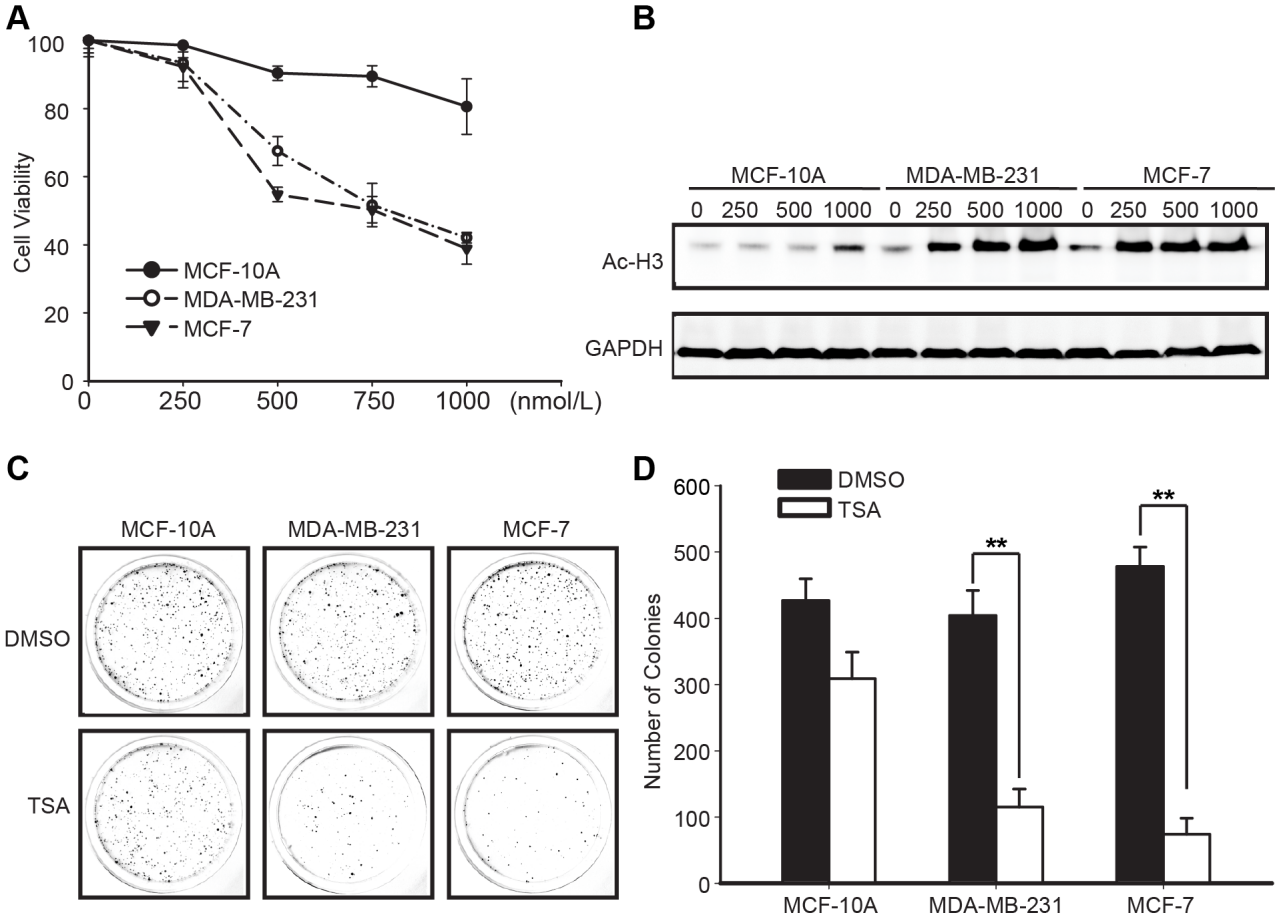
Effects of TSA on breast cancer cell viability and proliferation. (**A**) The effect of TSA on breast cancer cell viability. Exponentially growing cells were treated with the indicated concentrations of TSA for 48 h. Cell viability was assessed with an MTT assay. The MTT assay results were represented as percentages of viable cells, compared to the DMSO-treated cells. Graphs were shown as the means ± SD. (**B**) Acetylated H3 protein levels in MCF-10A, MDA-MB-231 and MCF-7 cells in response to a 48 h TSA concentration gradient. (**C, D**) Effect of TSA on breast cancer cell proliferation. Cell proliferation was determined with in vitro clonogenic assays. Representative images (C) and the numbers of colonies (**D**) indicated that TSA inhibited breast cancer cell growth. The histogram data showed the means ± SD of triplicate results. **P<0.01 versus DMSO (control) using Student's t-test.

We examined the effect of TSA on the viability of the MCF-10A, MCF-7, MDA-MB-231 cell lines with a MTT assay. After exposure to various concentrations of TSA for different time points (Figure S1A), the viabilities of the MCF-7 and MDA-MB-231 cell lines were reduced in a dose-dependent and time-dependent manner, with an IC_50_ of approximately 500 nmol/L at 48 h. The viability of the MCF-10A cell line was only decreased by 20% when the concentration of TSA was increased to 1000 nmol/L (Figure 1A). H3 acetylation of 500 nmol/L TSA for MCF-7, MDA-MB-231 and 1000 nmol/L for MCF-10A at different time points were visualized by western blotting. It was shown that the acetylation of H3 was apparent at 24 h and 48 h in breast cancer cells sparing non-transformed cells (Figure S1B). During treatment with serial concentrations of TSA, increasing H3 acetylation levels were observed in the MDA-MB-231 and MCF-7 breast cancer cells, but not in MCF-10A cell, indicating the TSA activity (Figure 1B). In a further investigation of the effect of TSA on breast cancer cell line proliferation, in vitro clonogenic assays showed that the numbers of colonies exposed to 500 nmol/L of TSA versus those in the DMSO control cultures were 115 versus 404 and 74 versus 478 in MDA-MB-231 and MCF-7 cell cultures, respectively, and 309 versus 407 in the MCF-10A cell cultures (Figure 1C, D). Collectively, these results indicated that TSA inhibited breast cancer cell viability and proliferation, but did not affect MCF-10A cell.

### Effects of TSA on cell cycle progression and apoptosis in breast cancer cells

**Figure 2:**
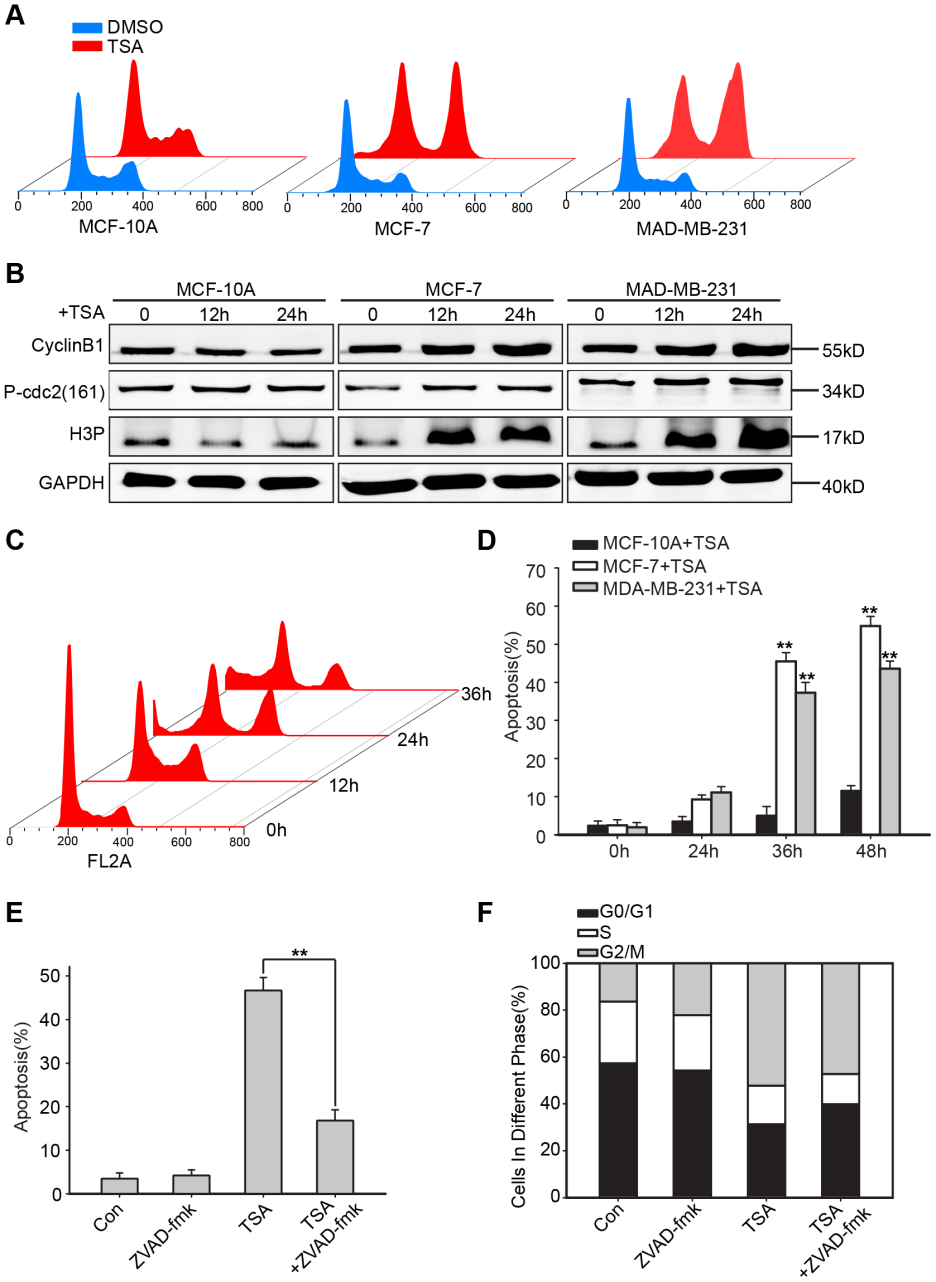
TSA caused G2-M phase cell cycle arrest and apoptosis in breast cancer cells. (**A**) Histograms depict cell cycle distributions in MCF-10A, MDA-MB-231 and MCF-7 cells after a 24 h treatment with DMSO (control) or TSA (1000 nmol/L, 500 nmol/L or 500 nmol/L). (**B**) Western blot of CyclinB1, Phospho-cdc2 (Thr161) and H3P in MCF-10A, MDA-MB-231 and MCF-7. Representative of 3 experiments. (**C**) Cell cycle distribution in MDA-MB-231 cells after exposure to 500 nmol/L of TSA at the indicated time points. (**D**) A time-course study of the effects of 1000 nmol/L, 500 nmol/L and 500 nmol/L TSA on the percentages of apoptotic cells in the MCF-10A, MDA-MB-231 and MCF-7 cell lines, respectively. Apoptosis was determined by flow cytometric analysis of the PI-positive and Annexin-V-positive cells. Values were shown as the means ± SD, n = 3. **P<0.01 versus the 0 h group using Student's t-test. A pan-caspase inhibitor, zVAD-fmk, was used to protect MDA-MB-231 cells from apoptosis; graphs showed the percentage of apoptotic cells under different treatments (**E**) and the cell cycle distributions (**F**). **P<0.01 compared to the TSA-treated alone group using Student's t-test.

To further examine the role of TSA in breast cancer cell growth inhibition, cell cycle progression and apoptosis were evaluated in flow cytometric analysis. MCF-10A, MCF-7 and MDA-MB-231 cells were treated with TSA (1000 nmol/L, 500 nmol/L and 500 nmol/L) for the indicated time points. MCF-7 and MDA-MB-231 cells exposure to 500 nmol/L of TSA for 24 h (Figure 2A) arrested in the G2-M phase, and the raw data was presented in Table S1. To further confirm the G2-M phase arrest, the expression of CyclinB1, Phospho-cdc2 (Thr161) and H3P was detected using western blotting. Enhanced expression of CyclinB1, Phospho-cdc2 (Thr161) and H3P in MCF-7 and MDA-MB-231 in response to TSA revealed the G2-M arrest (Figure 2B). The percentage of MDA-MB-231 cells arrested in the G2-M phase decreased, while subG1 phase cells began to accumulate after 24 h (Figure 2C). MCF-7 cell line showed similar results (data was not shown). To identify that the subG1 peak was due to apoptosis and not necrosis, we performed a flow cytometric apoptosis assay, using an Annexin V-FITC/PI kit. The percentages of apoptotic MCF-7 and MDA-MB-231 cells were minimal at 24 h (9.26% and 11.12%, respectively), but increased to 54.79% and 43.6%, respectively, at 48 h; MCF-10A cells were not affected (Figure 2D). Using the pan-caspase inhibitor zVAD-fmk to protect MDA-MB-231 cells from apoptosis, the G2-M arrest was not affected, although the percentage of TSA-mediated apoptotic cells was markedly reduced (Figure 2E,2F) and the raw data of cell cycle analysis of Figure 2F was supplemented with Table S2. In summary, TSA arrested breast cancer cell growth in the G2-M phase and induced apoptosis to inhibit the growth of transformed cells; the latter occurred after G2-M arrest.

### TSA triggered the intrinsic apoptosis pathway

**Figure 3:**
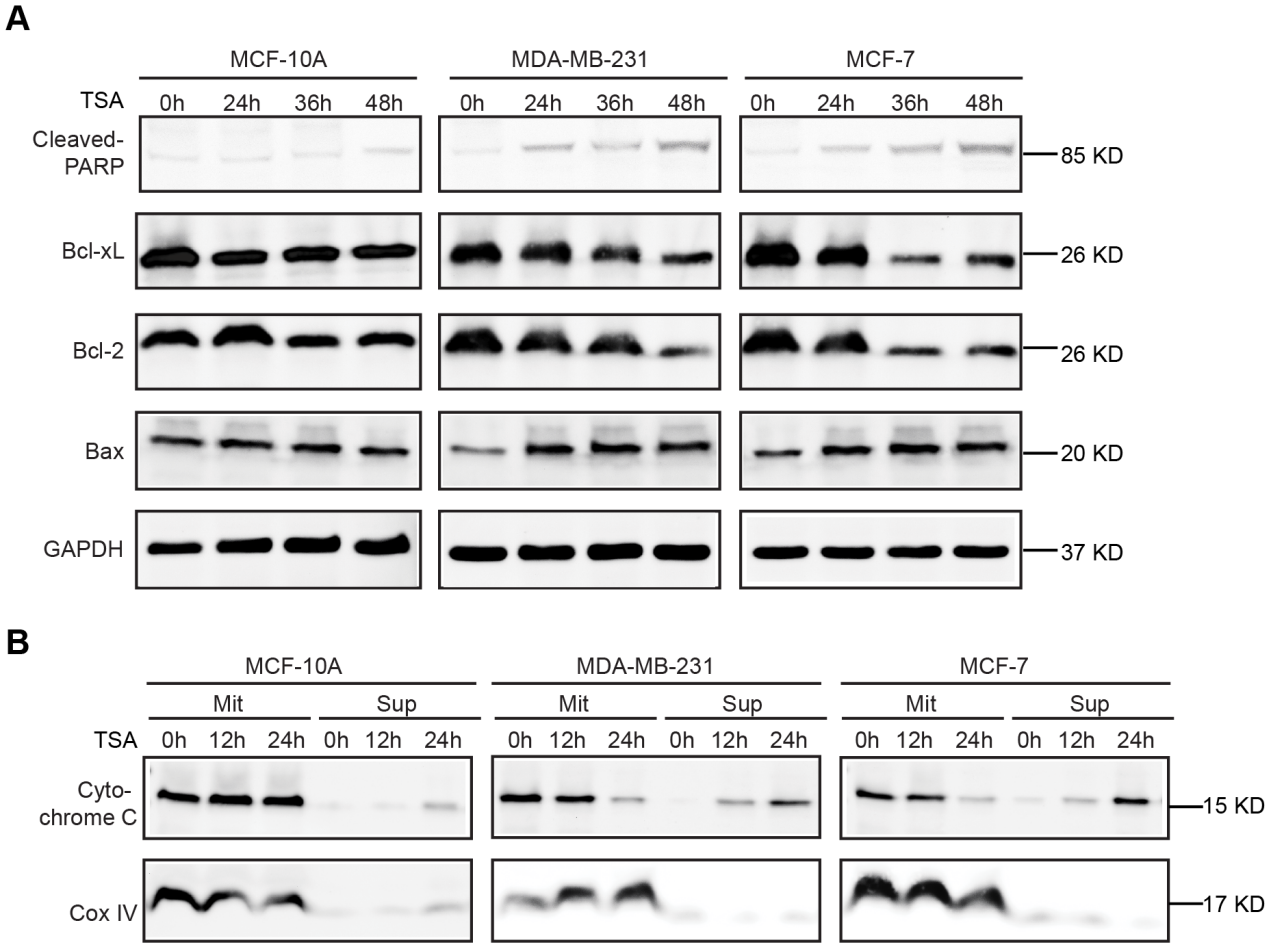
TSA triggered the intrinsic apoptosis pathway. (**A, B**) MCF-10A, MDA-MB-231 and MCF-7 cells were treated with 1000 nmol/L, 500 nmol/L and 500 nmol/L TSA respectively for the indicated time points. Western blotting was performed to evaluate cleaved-PARP, Bcl-2, Bcl-xL and Bax expression in cell lysates following exposure to the above-described treatment (**A**), and the resulting pellet (Mit) and supernatant (Sup) were immunoblotted for Cytochrome c and COX-IV (control; **B**). Similar results were observed in a total of 3 independent experiments.

The central events of HDACI-induced apoptosis involve the activation of pro-apoptotic BH3-only Bcl-2 family proteins and mitochondrial membrane disruption, leading to activation of the mitochondrial apoptosis pathway [9]. To confirm that TSA induced breast cancer cell apoptosis through the intrinsic pathway, we analyzed the expression of both anti-apoptotic (Bcl-2 and Bcl-xL) and pro-apoptotic (Bax) proteins by western blotting in the three cell lines. We also detected Cytochrome c localization in the mitochondrial and cytoplasmic fractions extracted from MCF-10A, MCF-7 and MDA-MB-231 cells. As shown in Figure 3A, TSA strongly downregulated Bcl-2 and Bcl-xL expression while inducing Bax expression in MCF-7 and MDA-MB-231 cells. TSA induced Cytochrome c release from the mitochondria into the cytoplasm in both breast cancer cell lines in a time-dependent manner, but had little effect on MCF-10A cells (Figure 3B).

### TSA-induced apoptosis was dependent on mitochondrial-derived ROS and reduced by antioxidants

**Figure 4:**
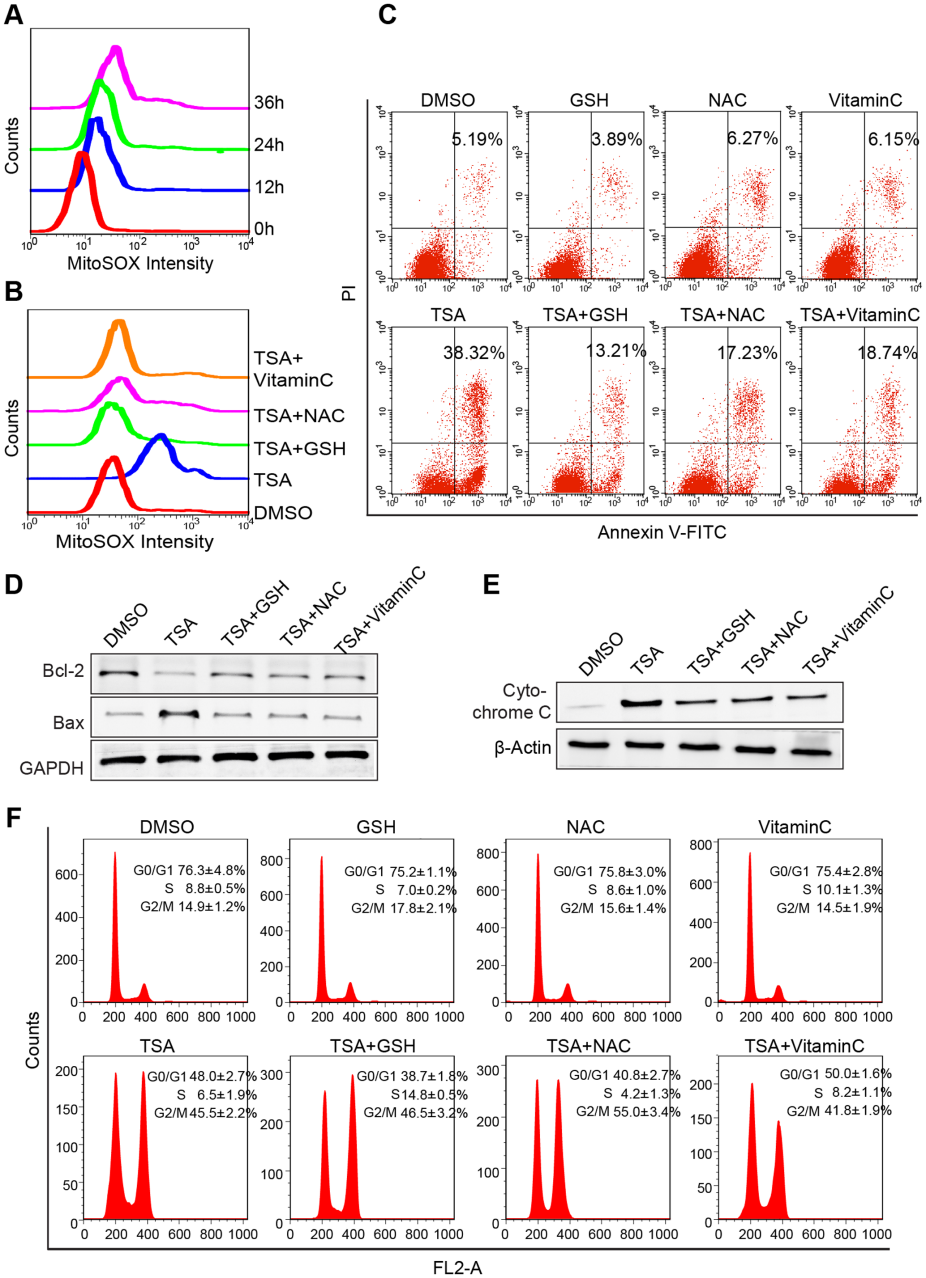
TSA-induced apoptosis was associated with mitochondrial-derived ROS and reduced by antioxidants treatment. (**A**) Time course of the Mito-SOX (highly selective indicator of superoxide in live cell mitochondria) fluorescence intensity in MDA-MB-231 treated 500 nmol/L of TSA detected by flow cytometry analysis. (**B**) Synergetic effects of antioxidants and TSA on mitochondrial-derived ROS in breast cancer cells. Exponentially growing cells were treated with DMSO (control) or 500 nmol/L of TSA with or without 10 mmol/L of NAC, 10 mmol/L GSH and 100 μmol/L Vitamin C for 36 h. The graph showed the Mito-SOX fluorescence intensity. (**C**) Synergetic effects of antioxidants and TSA on apoptosis in breast cancer cells. Graphs showed the percentages of PI-positive and Annexin-V-positive cells. (**D**) Immunoblotting of Bcl-2 family proteins in lysates from MDA-MB-231 cells that were cultured in the presence of the indicated treatments. (**E**) Cytochrome c release in indicated treated cells was analyzed by Western blotting. β-Actin, which was exclusively expressed cytosol, was used as control for loading and fractionation. (**F**) Synergetic effects of antioxidants and TSA on cell cycle in breast cancer cells. Graphs were shown as the means ± SD.

A previous study indicated that TSA induced the accumulation of reactive oxygen species (ROS), which might be important in HDACI-induced transformed cell death [20]. Thus, experiments were designed to explore the relationship between TSA-induced breast cancer cell apoptosis and mitochondrial reactive oxygen species (ROS). We used MDA-MB-231 cell line responses to TSA to represent the results, while MCF-7 cell line yielded similar results and the data was not shown. The generation of mitochondrial reactive oxygen species (ROS) in TSA-treated MDA-MB-231 cells at indicated time points was assessed by Mito-SOX staining, which is a highly selective detector of superoxide in live cell mitochondria, and flow cytometry. Consistent with TSA-induced breast cancer cell apoptosis, the intensity of Mito-SOX fluorescence was elevated in a time dependent manner, and increased by approximately 4.36 fold at 36 h (Figure 4A). Next, we examined whether mitochondrial ROS contributed to apoptosis. When administered with TSA, GSH, NAC and Vitamin C reduced the Mito-SOX fluorescence intensity by approximately 65.8%, 46.3% and 59.7% respectively in comparison to that in cells treated with TSA alone for 36 h (Figure 4B). Consistently, GSH, NAC and Vitamin C conferred resistance to TSA-induced apoptosis in MDA-MB-231 cells (Figure 4C), interfered with the TSA-mediated expression of Bcl-2 family proteins (Figure 4D), and protected Cytochrome C release from mitochondria to cytoplasm induced by TSA treatment (Figure 4E). However the G2-M phase arrest resulted from TSA in MDA-MB-231 was unaffected by GSH, NAC and Vitamin C (Figure 4F). In conclusion, these results suggested that TSA-mediated breast cancer cell apoptosis was dependent on mitochondrial-derived ROS production, which occurred upstream of the mitochondrial apoptosis pathway and could be blocked by antioxidants.

### TSA targeted the mitochondrial respiratory chain to induce apoptosis

**Figure 5:**
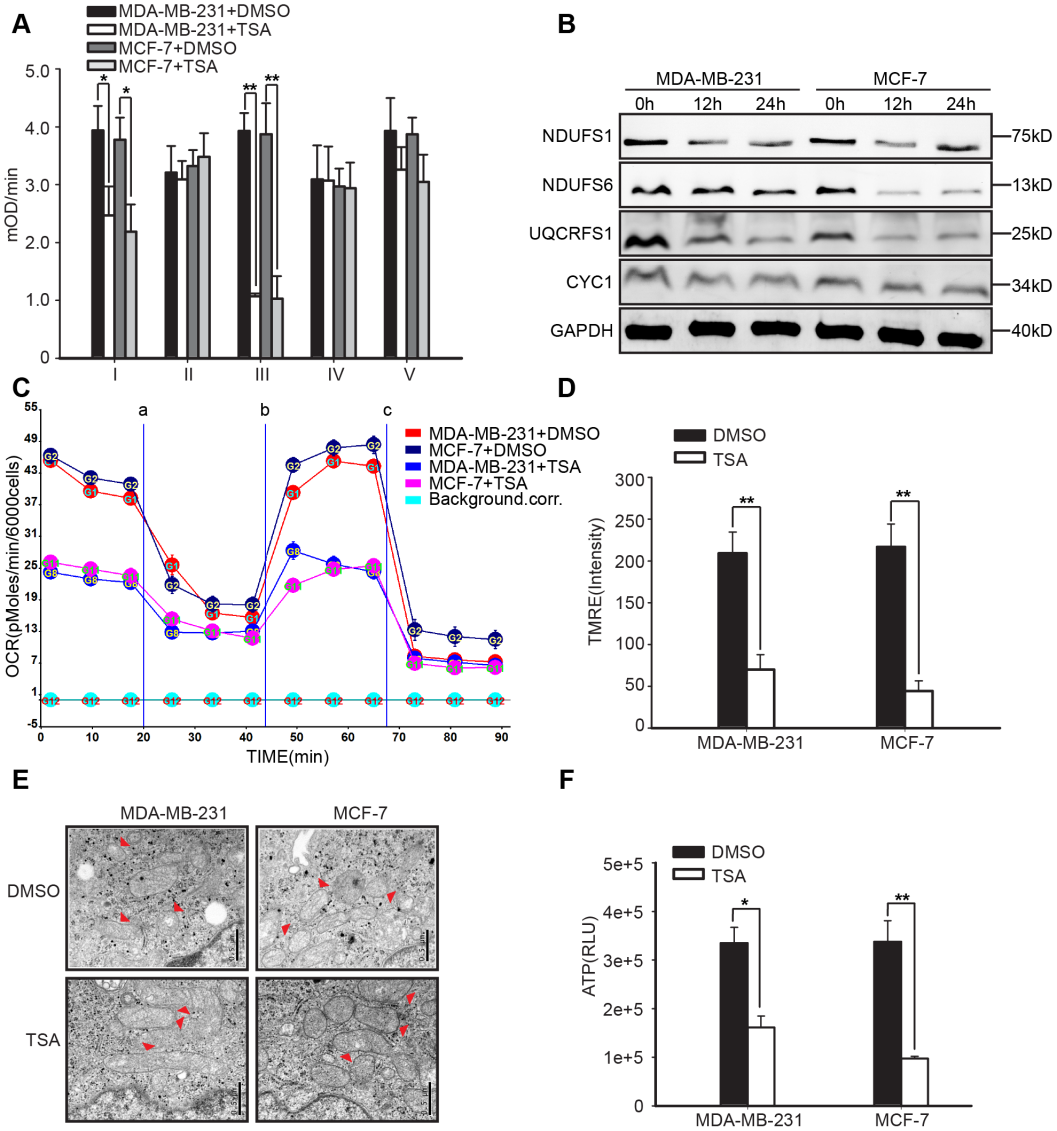
Impaired mitochondrial OXPHOS function in TSA-treated breast cancer cells. Exponentially growing MDA-MB-231 and MCF-7 cells were treated with DMSO (control) or 500 nmol/L of TSA. (**A**) Measurement of the mitochondrial complex I, II, III, IV and V activities exposure to TSA at 24 h (n = 3). Error bars indicated the SD; *P<0.05, **P<0.01 compared to DMSO treatment (control) using Student's t-test. (**B**) Expression of some subunits of mitochondrial complex I and complex III in response to TSA at 24 h. Representative of 3 experiments. (**C**) Oxygen consumption rate (OCR) in breast cancer cells treated with/without TSA for 24 h and sequentially exposed to (a) oligomycin, (b) FCCP, (c) rotenone and antimycin. Mean±SD, n = 5. (**D**) TMRE fluorescence as a measure of mitochondrial membrane potential (n = 3). Error bars indicated the SD; **P<0.01 compared to DMSO treatment in a Student's t-test. (**E**) Transmission electron microscopy showed differences in mitochondrial appearances. Δ indicated mitochondria. Scale bar  = 500 nm. (**F**) ATP content (n = 6). Error bars indicated the SD; *P<0.05, **P<0.01 compared to DMSO treatment (control) using Student's t-test.

Studies have reported that ROS generation is an inevitable by-product of mitochondrial oxidative metabolism [16]. To further characterize whether TSA impaired mitochondrial oxidative metabolism to enhance mitochondrial-derived ROS, we measured the activity levels of the oxidative phosphorylation (OXPHOS) complexes in DMSO-treated control and 500 nmol/L TSA-treated MDA-MB-231 cell and MCF-7 cell at 24 h. TSA markedly weakened the activity levels of complexes I and III, the main sources of mitochondrial-derived ROS in the mitochondrial respiratory chain, when compared with the DMSO control treatment (Figure 5A). To test this further, we examined whether the expression of some components of complexes I and III was altered by TSA. As observed in Figure 5B, the expression of NDUFS1, NDUFS6, which were two key subunits of complex I [21], and UQCRFS1, which played an essential role in complex III [22], was substantially reduced in response to TSA, while CYC1 did not alter markedly. We also detected the expression of NDUFV1, NDUFA9, UQCRC1, and UQCRC2 which showed little change (data not shown). To demonstrate this in detail, TSA reduced the expression of some components to weaken the activity of complex I and complex III, Therefore, oxygen consumption rate (OCR) was reduced more than 50% in response to 500 nmol/L TSA in breast cancer cells (Figure 5C). TSA-treated breast cancer cells presented with reduced TMRE fluorescence intensity at 36 h (Figure 5D), and electron microscopy demonstrated that TSA elongated the mitochondria with a higher resolution of the cristae, compared to the control-treated cells (Figure 5E). The impaired complex I and III activity, decreased TMRE fluorescence intensity and elongated mitochondria observed in the TSA-treated breast cancer cells suggested that the cells were undergoing accelerated cell death. Therefore, we measured the ATP levels in breast cancer cells after exposure to TSA for 36 h. The ATP levels in TSA-treated MDA-MB-231 and MCF-7 cells decreased by 51.8% and 71.2% (Figure 5F). Collectively, these data further reinforced our finding that TSA decreased the expression of some subunits of the complex I and III to impair their activity and induce mitochondrial derived ROS to activate mitochondrial apoptosis pathway.

## Discussion

It is increasingly apparent that the abnormal regulation of gene expression is a hallmark of tumorigenesis [23]. Aberrant regulation of gene expression via chromatin alteration seems to be a target for prevention and therapy, thus enriching the potential of histone deacetylase inhibitors (HDACIs) to be evaluated as leukemia and solid tumor treatments [24]. HDACIs exhibit various antitumor activities, including the induction of cell cycle arrest, differentiation and apoptosis [9]. The central event in HDACI-mediated cell death is activation of the intrinsic apoptotic pathway which involves the activation of pro-apoptotic BH3-only Bcl-2 proteins and mitochondrial membrane disruption [25]. However, the underlying mechanisms of this anti-tumor activity remain poorly defined. Several systems have illustrated that upon exposure to high doses of HDACI, cancer cells transitioned through the G1/S boundary and accumulated a 4N DNA content prior to apoptosis, while the overexpression of p16^INK4a^ to arrest cells in the G1 phase allowed cells to remain viable [26], [27]. HDACI-mediated tumor cell apoptosis was restored by the overexpression of Bcl2, although G2-M phase arrest was unaltered [28]. Hence, it is possible that the dysregulated mitosis might be the necessary event in HDACI-mediated apoptosis. Furthermore, recent studies on spindle poisons proposed a “competing-networks” model of cell fate. One network would maintain mitosis by escaping mitotic arrest, while the other would activate the apoptotic machinery. During a delay in mitosis, both networks would be active, and the network that eventually prevailed would determine the cell fate [29], [30]. Therefore, we postulated that the aberrant TSA-mediated mitosis activated apoptosis and thus determined the cell fate. Based on our study, we found that the antitumor activity of TSA in breast cancer cells was initiated by G2-M arrest and dependent on mitochondrial-mediated ROS through the inhibition of mitochondrial complex I and III activity. Following exposure to high doses of TSA, MCF-7 and MDA-MB-231 cells were arrested in the G2-M phase at 24 h, after which the proportion of arrested G2-M cells decreased and subG1 cells began to accumulate. Using a pan-caspase inhibitor, zVAD-fmk, to protect MDA-MB-231 cells from apoptosis, we found that G2-M arrest was unaffected, although the percentage of TSA-mediated apoptotic cells was markedly decreased. Hence, TSA-induced apoptosis may be initiated by G2-M phase arrest and might be the major necessary event for apoptosis induction. Recently accumulated evidence supports the finding that HDACIs enhanced ROS production [12], [13], [31], and the mitochondrial respiratory chain is the major source of ROS production [17], [18]. Thus, we hypothesized that TSA targeted the mitochondrial respiratory chain to induce ROS production and, eventually, apoptosis. As shown in the study, TSA enhanced mitochondrial-mediated ROS production and apoptosis in breast cancer cells, the rate of which was strongly reduced by antioxidants. However, the G2-M arrest resulted from TSA treatment was unaffected. Further findings illustrated that TSA weakened mitochondrial respiratory chain activity by decreased the expression of some subunits of complex I and complex III, which were the major sources of reactive oxygen species (ROS). This finding suggested that TSA targets the mitochondrial respiratory chain to enhance mitochondrial ROS production, disrupt the mitochondrial membrane potential and induce breast cancer cell death.

In conclusion, the present study established that TSA-induced apoptosis was initiated by G2-M arrest and was caused by enhanced mitochondrial-mediated ROS derived from reduced mitochondrial respiratory chain activity. This was the first study to bridge the relationship between the mitochondrial apoptosis pathway and mitochondrial metabolism. However, the mechanism of TSA's effects on mitochondrial metabolism should be explored further. As mitochondria have recently emerged as attractive cancer therapeutic targets, our findings might support the rationale for TSA alone or in combination as a novel therapeutic approach against breast cancer. Whether other chemotherapeutic drugs might also induce cell death by activating this pathway remains to be determined.

## Supporting Information

Figure S1 **TSA effects on viability in breast cancer cells.** (A) The effects of various concentrations of TSA at different time points on viability in the three cell lines. Graph was presented as mean± SD. (B) The expression of acetylated H3 in MCF-10A, MDA-MB-231 and MCF-7 cells in response to 500 nmol/L TSA at different time points. (Related to Figure 1).
(TIF)

Table S1 **The raw data of the cell cycle analysis.** (Related to Figure 2A).
(XLS)

Table S2 **The raw data of the cell cycle analysis.** (Related to Figure 2F).
(XLS)
