# Supplementary figures and images for: Trichostatin A Targets the Mitochondrial Respiratory Chain, Increasing Mitochondrial Reactive Oxygen Species Production to Trigger Apoptosis in Human Breast Cancer Cells
Source: PLoS One. 2014 Mar 13;9(3):e91610. doi: 10.1371/journal.pone.0091610 (PMC3953478; doi:10.1371/journal.pone.0091610)

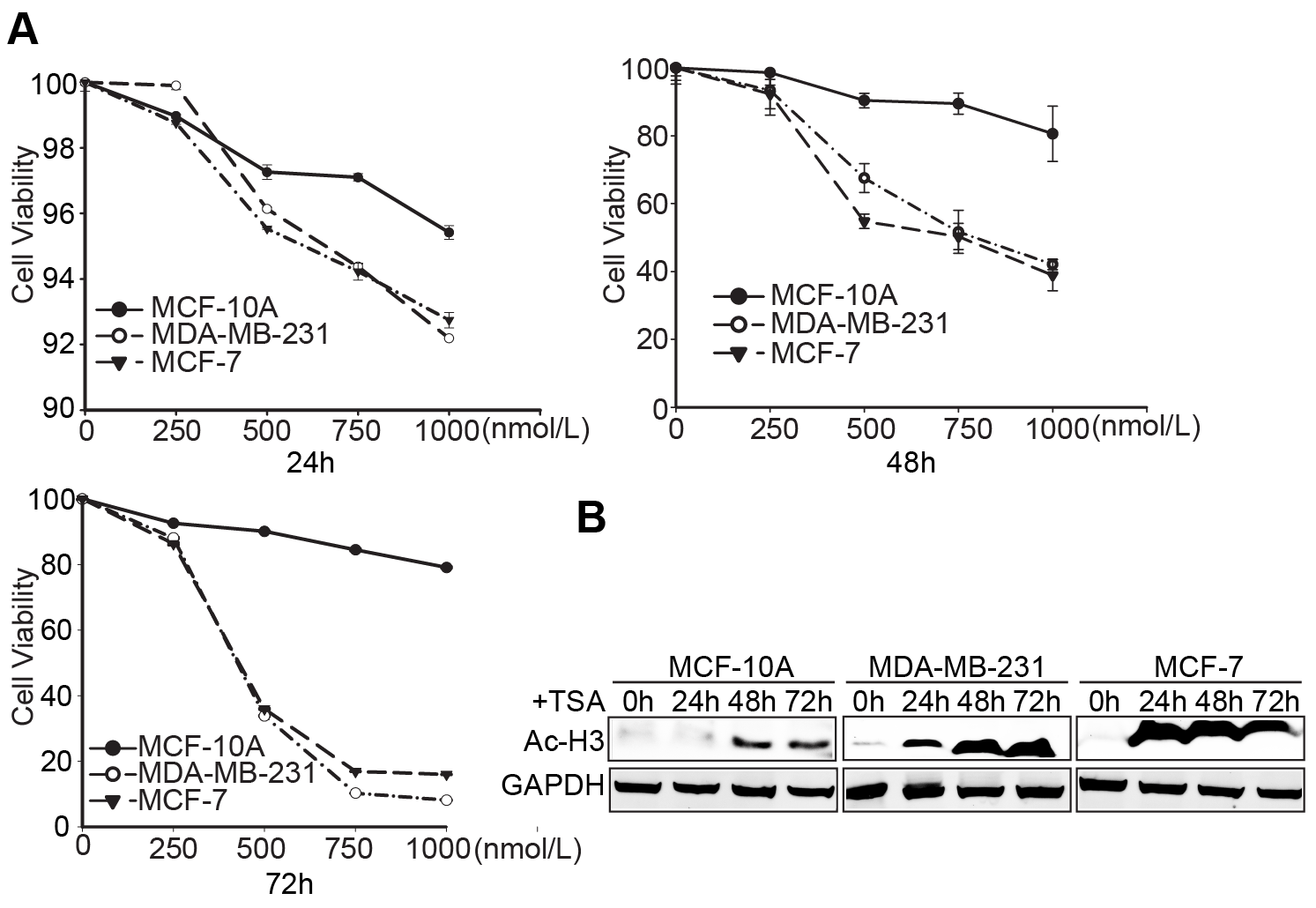

Supplement: Figure S1 — TSA effects on viability in breast cancer cells. (A) The effects of various concentrations of TSA at different time points on viability in the three cell lines. Graph was presented as mean± SD. (B) The expression of acetylated H3 in MCF-10A, MDA-MB-231 and MCF-7 cells in response to 500 nmol/L TSA at different time points. (Related to Figure 1). (TIF) [file pone.0091610.s001.tif]
